# An Engineered PfAgo with Wide Catalytic Temperature Range and Substrate Spectrum

**DOI:** 10.1002/advs.202416631

**Published:** 2025-05-14

**Authors:** Longyu Wang, Xiaochen Xie, Fuyong Huang, Qiang Wei, Tianxin Cai, Na Yu, Shi Chen, Fei Wang, Wanping Chen, Chin‐yu Chen, Chunhua Li, Lixin Ma

**Affiliations:** ^1^ State Key Laboratory of Biocatalysis and Enzyme Engineering, School of Life Sciences Hubei University Wuhan Hubei 430062 China

**Keywords:** cold adaptation, nucleases, PfAgo, rational engineering, thermophilic archaea

## Abstract

PfAgo, a thermophilic Argonaute nuclease from *Pyrococcus furiosus*, is widely used in various fields due to its high DNA‐guided DNA cleavage activity. However, its high‐temperature‐dependent cleavage activity largely restricts its applications in moderate‐temperature scenarios. In this study, PfAgo is engineered for cold adaptation based on its ternary complex structure and the attributes of cold‐adapted enzymes, yielding a series of variants with better performance at moderate temperatures. Among those, mPfAgo (K617G, L618G) exhibits significantly promoted cleavage activity at 37 °C and a wider catalytic temperature range of 30–95 °C. Its high‐temperature cleavage activity is also greatly improved, enabling its application in DNA detection with attomolar sensitivity in the presence of Mg^2+^. Additionally, mPfAgo shows versatile cleavage activities, including DNA cleavage guided by 5′OH‐gDNA, 5′P‐gDNA, or 5′COOH‐gDNA, as well as RNA cleavage with 5′OH‐gDNA, 5′P‐gDNA, 5′P‐gRNA, or 5′COOH‐gDNA as guides. Further analysis through far‐UV CD spectra and DSF indicates that mPfAgo has a more flexible structure than wild‐type PfAgo. Furthermore, this established strategy is applied to engineer TtdAgo, likewise obtaining its variants with enhanced moderate‐temperature activity and expanded substrate spectrum. In summary, this work provides a novel method for the rational design of thermophilic Agos, thereby greatly expanding their application scopes.

## Graphical abstract

This work presents a strategy for engineering thermophilic Ago proteins to obtain wide catalytic temperature range and broad substrate spectrum. This strategy is applied to engineer PfAgo and TtdAgo, and the biochemical properties of the engineered PfAgo are systematically characterized. The study further elucidates the structural differences involved. Overall, this work provides an alternate approach to obtaining desired Ago proteins.

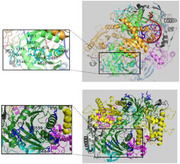

## 1 Introduction

Argonaute (Ago) proteins, an emerging class of programmable nucleases, employ small DNA/RNA guides (gDNA or gRNA) to cleave complementary nucleic acid targets. Unlike the widely used Cas nucleases, Ago nucleases do not rely on specific motifs in target molecules (such as PAM^[^ ^1^ ^]^ and PFS^[^ ^2^ ^]^) for their catalytic activity and use DNA/RNA as guides, offering the potential for development of various Ago‐based bio‐techniques. PfAgo, from the thermophilic archaea *Pyrococcus furiosus* and the most popular Ago nuclease, has been demonstrated high DNA‐guided cleavage activity toward both single‐ and double‐stranded DNA (dsDNA) targets at 87–99.9 °C,^[^ ^3^ ^]^ with the advantages of easier purification, preservation, and transportation due to its thermostability. A series of its applications, including in molecular cloning^[^ ^4^, ^5^ ^]^ and in nucleic acid detection,^[^ ^6^, ^7^, ^8^, ^9^, ^10^ ^]^ have been developed thereafter. While the high stability and low immunogenicity of PfAgo enable its potential in vivo application,^[^ ^11^, ^12^ ^]^ the low catalytic activity of PfAgo at moderate temperatures restricts its applications under mesophilic conditions. Recently, Zhou et al. applied a conditional protein diffusion model to generate artificial PfAgo sequences, which deviate by nearly 400 amino acids from their wild‐type templates, and the resulting proteins exhibited ssDNA cleavage activity at 45 °C.^[^ ^13^ ^]^

Although PfAgo is the first Ago protein whose crystal structure has been determined, neither guide nor target is included in the structure.^[^ ^14^ ^]^ In the early stage, we resolved the ternary complex structure of PfAgo binding with guide DNA and target DNA using cryo‐electron microscopy, and thereby gained an insight into its catalytic mechanism through structural and biochemical analysis.^[^ ^15^ ^]^ Similar to most Agos, PfAgo adopts a bi‐lobed architecture with the PAZ lobe (N domain, interdomain linker L1, and PAZ domain) connected by interdomain linker L2 to the PIWI lobe (MID and PIWI domains), among which the MID and PAZ domains respectively anchor the 5′ end and the 3′ end of guides during guide loading. The active center within the PIWI domain is structurally similar to that of RNase H,^[^ ^16^ ^]^ and the DEDH catalytic tetrad endows PfAgo with endonuclease activity.

Generally, enzymes from organisms in different environments maintain similar catalytic rates at their respective physiological temperatures, and the associated sequence variations with these adaptation differences are often in surface‐exposed areas distant from substrate‐binding sites, leaving the active site of an enzyme structurally unperturbed.^[^ ^17^, ^18^, ^19^ ^]^ Numerous studies reveal a common structural attribute of cold‐adapted enzymes, the presence of surface‐exposed glycine residues, which help increase both structural flexibility and enzymatic activity by acting locally near a catalytic site or through allosteric mechanisms.^[^ ^20^, ^21^, ^22^ ^]^

To improve the moderate‐temperature activity of PfAgo, we performed rational design based on its high‐resolution ternary structure and characteristics of cold‐adapted enzymes. A better‐performing variant named mPfAgo (K617G, L618G) was obtained with greatly improved cleavage activity at 37 °C. Compared to its wild‐type, mPfAgo exhibited a faster cleavage rate at high temperatures or in the presence of Mg^2+^, together with a broader temperature range of 30–95 °C for its cleavage activity. In addition, mPfAgo extended its substrate spectrum from DNA guide and DNA target to DNA/RNA guides and DNA/RNA targets. Further analysis of the *k*_cat_ values and the *K*_M_ values demonstrated higher cleavage efficiency as well as stronger affinity to substrates of mPfAgo, and results from far‐UV CD spectra and DSF measurement indicated a more flexible structure of mPfAgo, relative to wild‐type PfAgo. Moreover, mPfAgo achieved much higher DNA detection sensitivity with the detection limit as low as attomolar level in the presence of Mg^2+^, implying its superior application potential over wild‐type PfAgo. Furthermore, we applied this strategy to engineer another thermophilic Ago from *Thermococcus thioreducens*, TtdAgo,^[^ ^15^, ^23^ ^]^ likewise obtaining mutants with higher moderate‐temperature cleavage activity and a wider substrate spectrum. Thus, this study establishes a cold adaptation rational design strategy for thermophilic Agos and provides an alternate way to obtain desired Ago proteins.

## 2 Results and Discussion

### 2.1 Rational Engineering of PfAgo Yields a Mutant with High Moderate‐Temperature Activity and a Wide Substrate Spectrum

**Figure 1:**
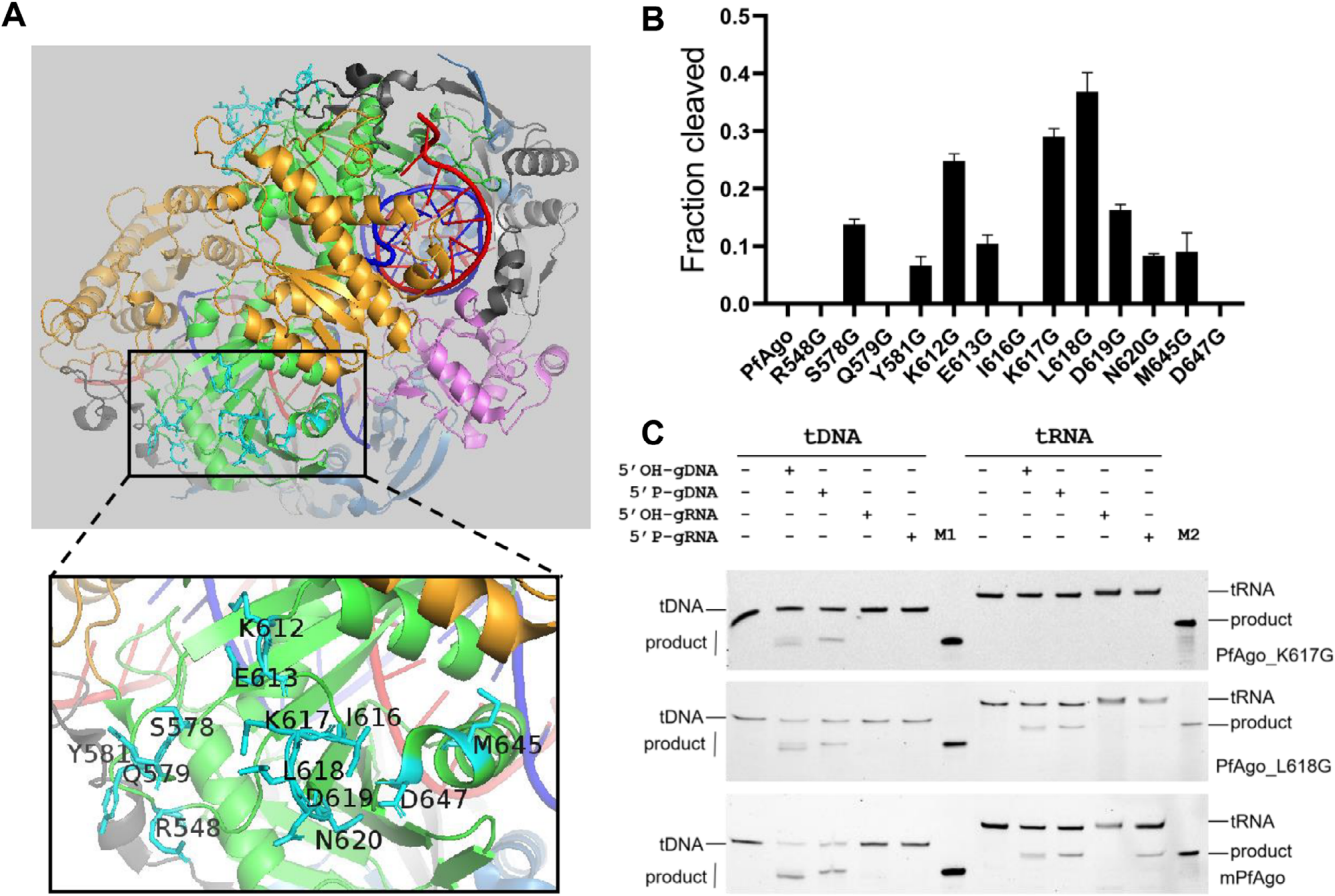
Rational engineering of PfAgo generates mutants with high cleavage activity at 37 °C. A) Structure of PfAgo ternary complex with the mutation sites marked. B) Quantitative comparison of DNA cleavage activity among wild‐type PfAgo and its mutants at 37 °C (*n* = 3). Data are represented as the mean ± SD. C) Cleavage activity assay of PfAgo mutants at 37 °C using 5′FAM‐labeled DNA and RNA as targets, respectively. M1 indicates synthesized 5′FAM‐labeled, 34‐nt DNA product. M2 indicates synthesized 5′FAM‐labeled, 34‐nt RNA product.

To enhance the cleavage activity of PfAgo at moderate temperatures, we performed structure‐guided rational design of PfAgo for cold adaptation with the following four principles: 1) the side chain of the mutation site is highly exposed to the surface with no intramolecular contact; 2) the side chain of the mutation site is far away from the active site and the bound substrate (i.e., >20Å); 3) the mutation site is located in the PIWI domain; 4) the mutation residue is substituted with glycine. The bound substrates here refer to guide and target nucleic acids, and the active site refers to the DEDH catalytic tetrad. The structure of the PfAgo ternary complex (PfAgo‐guide DNA‐target DNA, PDB ID: 8JPX) was analyzed with PyMOL software, and thirteen sites were chosen to be substituted with glycine, respectively (**Figure** **1**A). Cleavage of target DNA guided with 5′P‐gDNA was then performed at 37 °C, respectively. The results showed that nine of the thirteen mutants were catalytically active at 37 °C, and among them, two mutants, PfAgo_K617G and PfAgo_L618G, exhibited much higher cleavage activity (Figure 1B; Figure S1A, Supporting Information).

To determine the types of guides and targets of PfAgo_K617G and PfAgo_L618G at 37 °C, four types of guides (5′OH‐gDNA, 5′P‐gDNA, 5′OH‐gRNA, and 5′P‐gRNA) were used for cleavage of complementary target DNA or RNA. The results showed apparent cleavage activity of both PfAgo_K617G and PfAgo_L618G toward DNA target guided by 5′OH‐gDNA and 5′P‐gDNA at 37 °C. Unexpectedly, cleavage of the complementary RNA target was detected with PfAgo_L618G guided by 5′OH‐gDNA or 5′P‐gDNA at 37 °C. 5′P‐gRNA‐mediated cleavage of the RNA target by PfAgo_L618G was observed as well, albeit less active. Subsequently, combination of these two mutations yielded a more active mutant called mPfAgo (K617G/L618G), which exhibited much higher cleavage activity at 37 °C toward DNA target guided by either 5′OH‐gDNA or 5′P‐gDNA, as well as RNA target by 5′OH‐gDNA, 5′P‐gDNA, or 5′P‐gRNA, compared to either of these two single mutants (Figure 1C). In addition, mPfAgo can employ 5′COOH‐gDNA to guide cleavage of target DNA and RNA (Figure S1B, Supporting Information). Together, these results indicated that the engineered mPfAgo gained an intensively enhanced moderate‐temperature catalytic activity, coupled with an unexpected wider substrate spectrum.

### 2.2 mPfAgo Possesses DNA‐guided RNA Cleavage Activity

**Figure 2:**
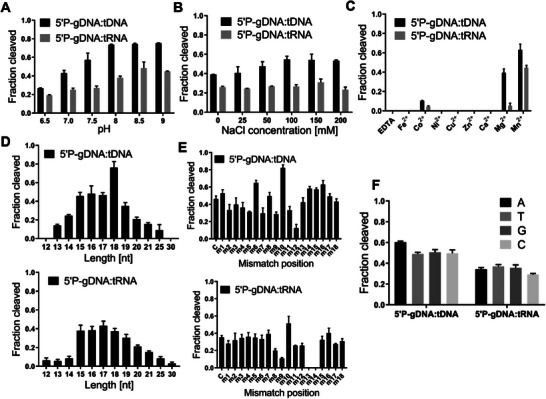
mPfAgo possesses DNA‐guided DNA and RNA cleavage activity at 37 °C. Effects of the pH A), NaCl concentration B), divalent metal ion C), guide length D), guide‐target mismatch E) and 5′‐end nucleotide of guide F) on DNA and RNA cleavage activity of mPfAgo with 5′P‐gDNA were inspected, respectively, at 37 °C (*n* = 3). Data are represented as the mean±SD.

PfAgo has an innate ability to cleave target DNA guided by DNA. However, mPfAgo shows DNA‐guided DNA and RNA cleavage activity at 37 °C. To systematically assess the DNA‐guided cleavage activity of mPfAgo at 37 °C, we measured the cleavage activity of mPfAgo toward target DNA and RNA under different conditions. In general, the optimal reaction conditions for an enzyme are related to the growth environment of its native host. We first examined cleavage of target DNA or RNA by mPfAgo guided by 5′P‐gDNA at varied pH values or NaCl concentrations. Whether DNA or RNA target, mPfAgo showed efficient cleavage activity at pH 8–9 (**Figure** **2**A; Figure S1C, Supporting Information). Meanwhile, mPfAgo showed higher activity toward DNA target at the NaCl concentrations of 50–150 mm, but similar cleavage activity toward RNA target at 0–200 mm of NaCl (Figure 2B; Figure S1C, Supporting Information). Considering the essential role of divalent metal ions in the binding of 5′P‐guides to and the catalytic activity of Ago proteins,^[^ ^24^ ^]^ we next tested the 5′P‐DNA‐guided cleavage activity of mPfAgo toward target DNA or RNA in the presence of different divalent metal ions. Cleavage of both DNA and RNA targets was detected in the presence of Co^2+^, Mg^2+,^ or Mn^2+^, but more efficient cleavage was observed with Mn^2+^ (Figure 2C; Figure S1D, Supporting Information). We then further investigated the effects of different Mn^2+^ or Mg^2+^ concentrations on this cleavage activity. The results showed cleavage of both DNA and RNA targets across the whole Mg^2+^ concentration range of 1–10 mm but relatively less effective toward RNA target, with the highest DNA cleavage activity at 10 mm (Figure S2A,B, Supporting Information). However, comparable DNA or RNA cleavage activity was observed over the whole Mn^2+^ concentration range of 1–10 mm (Figure S2A,C, Supporting Information).

In addition, we examined the effects of 5′P‐gDNA lengths on the DNA or RNA cleavage activity of mPfAgo. 5′P‐gDNAs of 12–30 nt lengths with identical 5′‐terminal sequences were employed in the cleavage experiments. The results showed that 5′P‐gDNAs of 13–25 nt lengths guided cleavage of target DNA with the highest activity by the 18‐nt‐long one. For cleavage of RNA target, all adopted 5′P‐gDNAs guided active cleavage with better cleavage by those of 15–18 nt lengths (Figure 2D; Figure S3A, Supporting Information). To characterize the 5′P‐DNA‐guided cleavage specificity of mPfAgo, we tested the impacts of guide/target mismatches on the DNA and RNA cleavage activity. In the effector complex of Agos, the guide molecule can be functionally divided into several regions, including the 5′‐end region (position 1), 5′‐seed region (positions 2–8), central region (positions 9–12), 3′‐supplementary region (positions 13–15), and 3′‐tail region (positions 16–18).^[^ ^25^ ^]^ The specificity of mPfAgo varied depending on the target. As shown in Figure 2E; Figure S3B (Supporting Information), single mismatches at positions 2, 3, 4, 5, or 7 in the 5′‐seed region of 5′P‐gDNA impaired the cleavage of DNA target, but only the single mismatch at position 8 decreased the cleavage of RNA target. Besides, either DNA or RNA target cleavage was inhibited by the single mismatches at positions 9, 11, or 12 in the central region of 5′P‐gDNA. However, the single mismatch at positions 13 or 14 in the 3′‐supplementary region of 5′P‐gDNA deteriorated merely through cleavage of RNA target but not the DNA target. Moreover, the single mismatch at nucleotide 12 of 5′P‐gDNA heavily reduced DNA cleavage, and those at nucleotide 13 or 14 completely blocked RNA cleavage. Instead, the single mismatch at position 10 of 5′P‐gDNA increased both the DNA and RNA cleavage activity of mPfAgo.

The preference for the 5′‐end nucleotide of guides has been seen in many Ago proteins and in many respects, such as in binding of endogenous nucleic acids in vivo,^[^ ^26^ ^]^ the structure‐based binding of guides,^[^ ^27^, ^28^ ^]^ and the in vitro cleavage activity of Ago proteins.^[^ ^29^, ^30^ ^]^ To investigate the influences of the 5′‐end nucleotide of 5′P‐gDNA on the DNA and RNA cleavage activity of mPfAgo, we synthesized a series of 5′P‐gDNA with different 5′‐end nucleotides but otherwise identical sequences and subjected them to investigation. The results showed a slightly higher DNA cleavage activity by 5′P‐gDNA with 5′‐A than with 5′‐T, 5′‐G, or 5′‐C, whereas no obvious preference for the 5′‐end nucleotide during RNA cleavage (Figure 2F; Figure S3C, Supporting Information).

To assess the robustness of mPfAgo in biologically relevant contexts, we examined its cleavage activity in bacterial and mammalian cell lysates at 37 °C, respectively. The results showed significant cleavage of target DNA, demonstrating the robust specific cleavage activity of mPfAgo even in these complex environmental systems (Figure S3D, Supporting Information).

Together, these results demonstrated that mPfAgo acquired specific and efficient 5′P‐DNA‐guided DNA and RNA cleavage activity at 37 °C, albeit a bit lower activity toward RNA target.

### 2.3 mPfAgo Possesses RNA‐guided RNA Cleavage Activity

**Figure 3:**
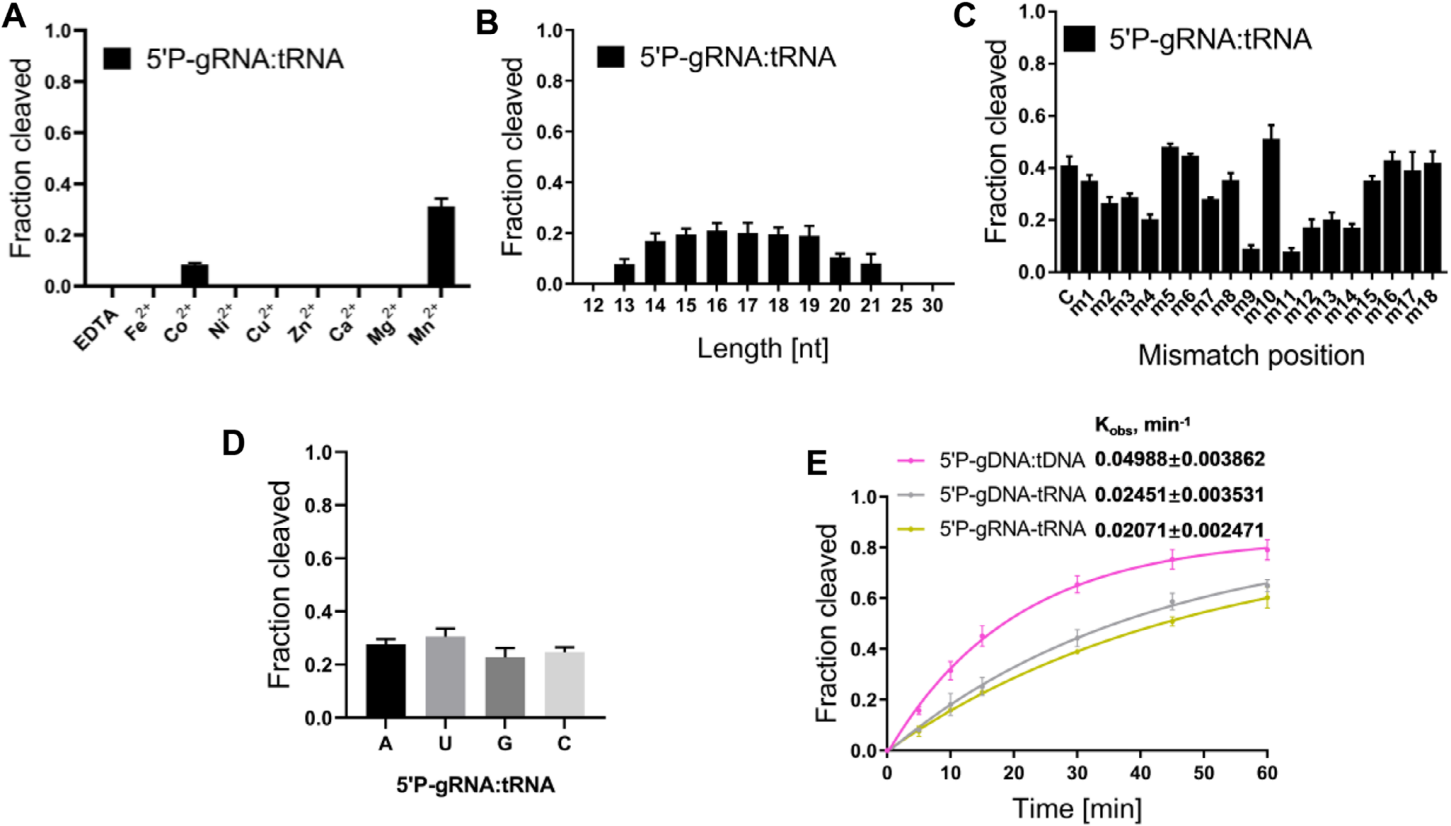
mPfAgo possesses RNA‐guided RNA cleavage activity at 37 °C. Effects of the divalent metal ion A), guide length B), guide‐target mismatch C) and 5′‐end nucleotide of guide D) on RNA cleavage activity of mPfAgo with 5′P‐gRNA were inspected, respectively, at 37 °C (*n* = 3). Data are represented as the mean ± SD. E) Kinetics of target cleavage by mPfAgo at 37 °C (*n* = 3). Data are fitted to a single exponential equation, and the resulting *k*_obs_ value is shown for each guide‐target pair.

PfAgo belongs to the clade of euryarchaeal thermophilic Agos, a prokaryotic Ago clade that is most closely related to eukaryotic Ago (eAgo) proteins, and generally prefers DNA guide for DNA target cleavage,^[^ ^3^ ^]^ while eAgo proteins usually identify RNA target using RNA guide.^[^ ^31^ ^]^ Through cold‐adaption rational engineering, mPfAgo unexpectedly acquired RNA‐guided RNA cleavage activity. We then further investigated this enzymatic attribute. The results showed that mPfAgo used only Co^2+^ or Mn^2+^ as divalent metal ions for its 5′P‐RNA‐guided RNA cleavage activity, and Mn^2+^ gave higher cleavage activity (**Figure** **3**A; Figure S1D, Supporting Information). Meanwhile, we examined the 5′P‐RNA‐guided RNA cleavage activity of PfAgo and mPfAgo at high temperatures in the presence of Co^2+^ or Mn^2+^ and found that mPfAgo, rather than PfAgo, was active (Figure S2D, Supporting Information). Further Mg^2+^ titration experiment showed that no obvious cleavage was observed even when the Mg^2+^ concentration was increased to 10 mm (Figure S2A,B, Supporting Information). Mn^2+^ titration experiment showed that mPfAgo was active at 1–10 mm of Mn^2+^, with higher activities over the concentration range of 3–10 mm (Figure S2A,C, Supporting Information).

In addition, we synthesized 5′P‐gRNAs of 12–30 nt lengths with identical sequences at their 5′ ends and examined their respective influences on the cleavage activity of mPfAgo. The results showed effective cleavage of target RNA guided by 5′P‐gRNA of 13–21 nt lengths, while better cleavage by those of 14–19 nt lengths (Figure 3B; Figure S3A, Supporting Information). Influences of guide/target mismatches on the 5′P‐RNA‐guided cleavage activity of mPfAgo were determined as well, and a series of 5′P‐gRNA with single mismatches at different sites were synthesized. Results of cleavage experiments showed that single mismatches at sites 2, 3, 4, or 7 in the 5′‐seed region, sites 9, 11, or 12 in the central region and sites 13 or 14 in the 3′‐supplement region of guides reduced the cleavage activity of mPfAgo, with seriously decreased cleavage activity detected at the sites 9 or 11 in the central region of guides. Conversely, single mismatches in the 5′‐end or 3′‐tail region of guides exerted little effect on the cleavage activity, and the single mismatch at site 10 in the central region of guides increased the cleavage activity (Figure 3C; Figure S3B, Supporting Information). In the meantime, we synthesized a series of 5′P‐gRNA with different 5′‐end nucleotides but otherwise identical sequences and examined their impacts on the cleavage activity. No obvious differential catalytic activity was observed, manifesting no apparent preference of mPfAgo for 5′‐end nucleotide of 5′P‐gRNA (Figure 3D; Figure S3C, Supporting Information). Altogether, these results demonstrated that mPfAgo acquired the specific 5′P‐RNA‐guided RNA cleavage activity via rational engineering, suggesting that PfAgo may be a vital intermediator during the evolution journey of Agos.

With these optimized cleavage conditions in hand, DNA and RNA cleavage kinetics of mPfAgo were measured at 37 °C using 5′P‐DNA or 5′P‐DNA/5′P‐RNA as guides, respectively. The fastest reaction rate was observed for 5′P‐gDNA‐mediated DNA cleavage with the *k*_obs_ value of 0.04988±0.003863, followed by 5′P‐gDNA and 5′P‐gRNA‐mediated RNA cleavage, with the respective *k*_obs_ values of 0.02451±0.003531 and 0.02071±0.002471 (Figure 3E; Figure S4A, Supporting Information).

### 2.4 mPfAgo Displays a Wide Catalytic Temperature Range and Enhanced Activity at High Temperatures

**Figure 4:**
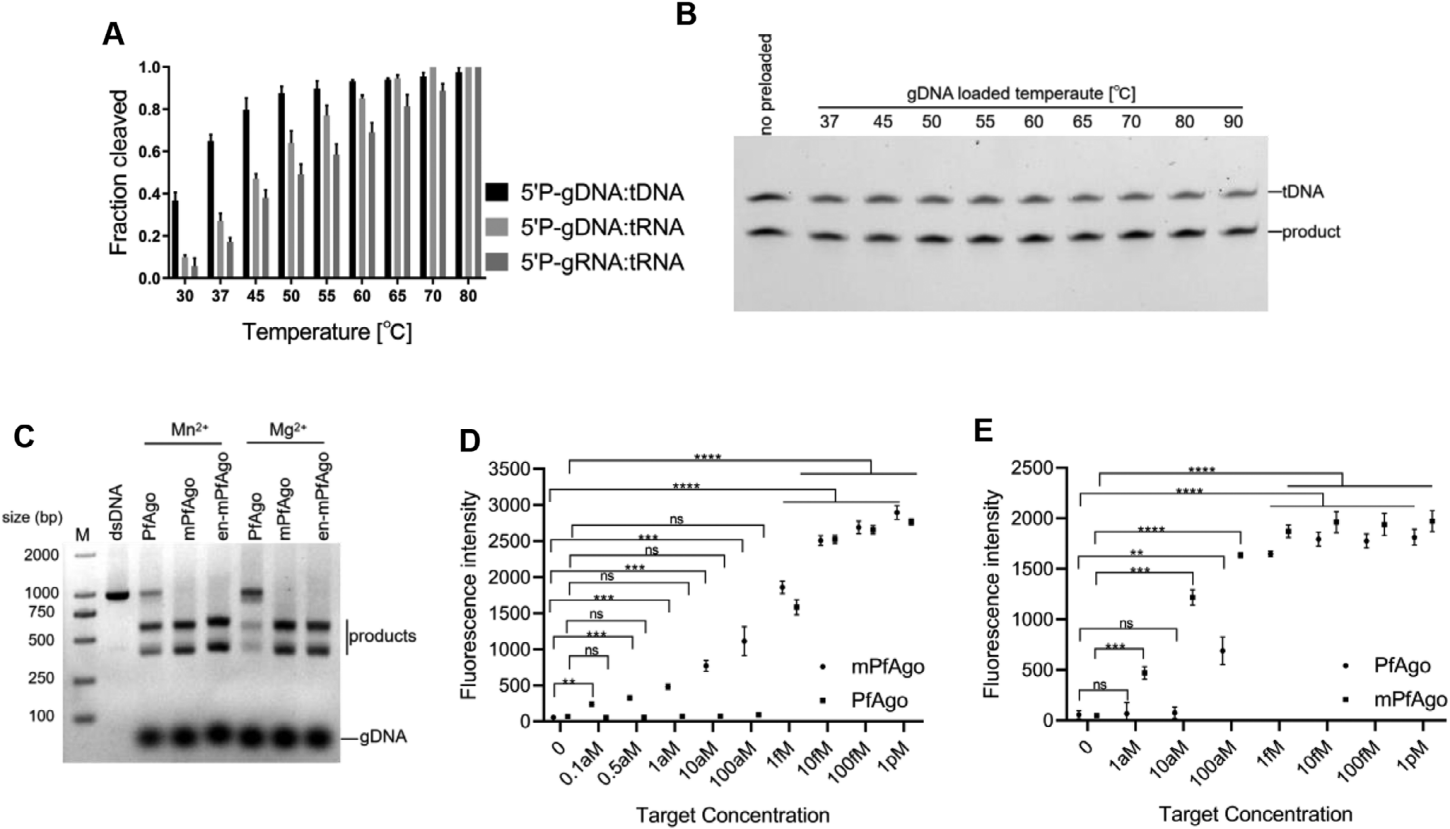
mPfAgo displays a wide catalytic temperature range and enhanced activity at 95 °C. A) Effects of reaction temperature on the mPfAgo cleavage activity (*n* = 3). Data are represented as the mean ± SD. B) The gel diagram showing target DNA cleavage at 37 °C by mPfAgo with different gDNA loaded temperatures. C) The dsDNA cleavage activity of PfAgo and its mutants at 95 °C. M, molecular weight marker; gDNA, gDNA‐F/gDNA‐R. The size of substrates is 1 kb. The size of products is 609 and 391 bp. D) The HPV16E6 gene detection combing PfAgo/mPfAgo with PCR in the presence of Mg^2+^ (*n* = 3). E) The *swp* gene detection combing PfAgo/mPfAgo with RPA in the presence of Mg^2+^ (*n* = 3). Data are represented as the mean ± SD. A value of *p* < 0.05 was considered to be statistically significant (*p < 0.05, **p < 0.01, ***p <0.001, and ****p < 0.0001).

To explore the catalytic temperature range of mPfAgo, we performed 5′P‐DNA‐guided DNA/RNA cleavage as well as 5′P‐RNA‐guided RNA cleavage at distinct temperatures. The results showed that mPfAgo displayed cleavage activity at a wide temperature range of 30–80 °C (**Figure** **4**A; Figure S4B, Supporting Information). Maybe due to the instability of target RNA at 90 °C, only cleavage of DNA target at 90 °C was observed (Figure S4B, Supporting Information). To explore the heat resistance of mPfAgo, we premixed mPfAgo with 5′P‐gDNA at different temperatures and tested its cleavage activity at 37 °C. The results showed that mPfAgo still preserved high cleavage activity at 37 °C even if the premixed temperature was increased to 90 °C (Figure 4B).

Thereafter, we further compared the high‐temperature cleavage activity toward DNA target between mPfAgo and PfAgo. The results showed a much higher cleavage rate of mPfAgo than that of wild‐type PfAgo in the presence of Mg^2+^ (Figure S5A, Supporting Information). Then, their respective substrate turnover kinetics at 95 °C were investigated in the presence of Mg^2+^ or Mn^2+^, and mPfAgo yielded much higher turnover rate than its wild type under all tested conditions (Figure S5B, Supporting Information). The dsDNA cleavage activity of PfAgo and mPfAgo at 95 °C was further tested, and mPfAgo showed significantly higher dsDNA cleavage activity in the presence of Mg^2+^ (Figure 4C). From the application viewpoint, the high cleavage activity of mPfAgo in the presence of Mg^2+^ would enable, on the one hand, the one‐pot reaction in nucleic acid detection, and on the other hand, low risk of non‐specific degradation of nucleic acids in both gene assembly and nucleic acid detection probably caused by Mn^2+^.^[^ ^32^ ^]^ Therefore, we implemented target DNA detection with PfAgo and mPfAgo in the presence of Mg^2+^ (Figure S5C, Supporting Information), and found that mPfAgo achieved much higher sensitivity with attomolar detection limit (Figure 4D,E). In addition, we conducted quantitative analysis on the catalytic performance of wild‐type PfAgo and mPfAgo at 95 °C by virtue of the Michaelis‐Menten kinetics model. The results showed a higher *k*_cat_ value and a lower *K*_M_ for mPfAgo than those for wild‐type PfAgo, respectively, with the *k*_cat_/*K*_M_ value for mPfAgo nearly 2.4 times that for wild‐type PfAgo (Figure S5D, Supporting Information), indicating both a higher catalytic efficacy and a stronger affinity to substrate for mPfAgo, compared to wild‐type PfAgo.

### 2.5 The Structure of mPfAgo is More Flexible

**Figure 5:**
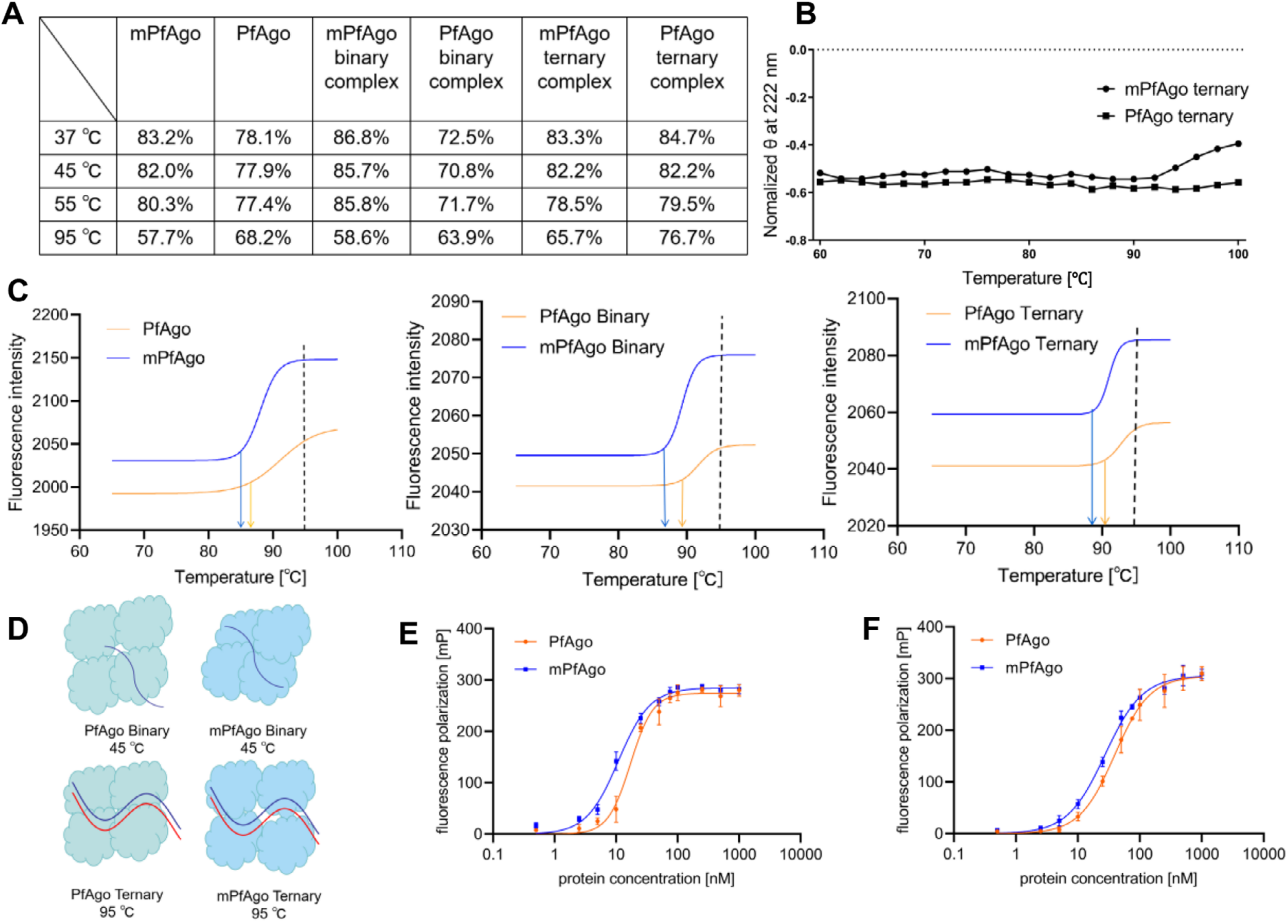
The structure of mPfAgo is more flexible than that of wild‐type PfAgo. A) Comparison of the α‐helix content between PfAgo and mPfAgo at different temperatures. The α‐helix content was analyzed with CDNN software following the far‐UV CD spectra measurement. B) Thermal unfolding curves of PfAgo/mPfAgo ternary complex measured by far‐UV CD spectroscopy. C) Thermal unfolding curves of PfAgo and mPfAgo through DSF measurement (*n* = 3). The fluorescence intensity is plotted against temperature and fitted using the Boltzmann sigmoidal model. The solid arrows and dashed lines represent *T*_on_ and *T*_phy_, respectively. D) A cartoon diagram illustrating the structural compactness of PfAgo and mPfAgo. The blue and red curves represent the guide and target, respectively. E) Binding analysis of 5′P‐gDNA by PfAgo and mPfAgo (*n* = 3). PfAgo and mPfAgo bind 5′P‐gDNA with average *K*_D_ values of 17.13 ± 1.91 and 10.87 ± 1.02 nm, respectively. F) Binding analysis of the 5′P‐gDNA:tDNA duplex by PfAgo and mPfAgo (*n* = 3). PfAgo and mPfAgo bind the 5′P‐gDNA:tDNA duplex with average *K*_D_ values of 39.27 ± 4.96 and 27.42 ± 2.56 nm, respectively. The fluorescence polarization is plotted against protein concentration and fitted using the model of specific binding with the Hill slope.

To gain more insights into the differential cleavage activity between mPfAgo and its wild type, the temperature dependence of their respective secondary structure was analyzed using far‐UV circular dichroism (CD) spectroscopy (Figure S6, Supporting Information). As shown in **Figure** **5**A, binding of guides with either mPfAgo or PfAgo gave rise to an apparent change in the content of α‐helix over the whole thermal process, and so did binding of target to the binary complex, indicating obvious change in their secondary structure. Moreover, more significant differences in the α‐helix percentage between their binary complexes were observed, with much higher α‐helix content in the binary complex of mPfAgo than that of PfAgo at moderate and moderate‐to‐high temperatures. It is likely that higher α‐helix content of mPfAgo contributes to stabilizing its binary complex in a catalytically favorable configuration, thereby favoring its cleavage activity at moderate temperatures. In contrast, much lower α‐helix content of mPfAgo than that of PfAgo was detected at 95 °C either in an apo‐enzyme or in complexes. Since being relatively loosely packed at this temperature, mPfAgo might adjust to lower α‐helix content to allow higher flexibility available for its higher cleavage activity than that of PfAgo at 95 °C (Figure 5A,B; Figure S7, Supporting Information). These results indicated higher structural stability at moderate temperatures as well as better structural flexibility at high temperatures of mPfAgo, enabling mPfAgo promoted cleavage activity at both moderate and high temperatures.

In the meantime, we conducted differential scanning fluorimetry (DSF) measurements to monitor the tertiary structure alteration for the engineered mPfAgo and its wild type over the thermal unfolding process. *T*_phy_ (physiological temperature) was observed higher than *T*_on_ (onset temperature of thermal unfolding) for both PfAgo and mPfAgo (Figure 5C), which was in accordance with the report by Zheng et al.^[^ ^33^ ^]^ and indicated partially disrupted tertiary structures for both of them under the physiological conditions, thus conferring them great structural flexibility and the ensuing high catalytic activity. Moreover, apo‐mPfAgo or mPfAgo binary/ternary complexes all showed lower *T*_on_ than those of PfAgo (Figure 5C), indicating more unfolded tertiary structure and higher structural flexibility of mPfAgo, which accordingly gave rise to slightly lower *T*_m_ for both apo‐mPfAgo and its binary/ternary complex (Figure S7C, Supporting Information). These structural superiorities of mPfAgo over PfAgo may contribute to its higher catalytic activity, whereas the preserved similar thermostability as that of PfAgo is likely due to the subtleness of structural differences between them (Figure 5D). Furthermore, a fluorescence polarization assay was performed at 37 °C to measure the substrate affinity of PfAgo and mPfAgo. The results showed a slightly lower dissociation constant (*K*_D_) value of 10.87 ± 1.02 nm for mPfAgo/gDNA complex than that for PfAgo/gDNA (*K*_D_: 17.13 ± 1.91 nm) (Figure 5E). Likewise, binding of the gDNA:tDNA duplex to mPfAgo was found to be slightly stronger than that to PfAgo, with the respective *K*_D_ value of 27.42 ± 2.56 and 39.27 ± 4.96 nm (Figure 5F). These results demonstrated a slightly stronger affinity of mPfAgo to substrates at 37 °C compared to PfAgo, which may be one of the factors contributing to mPfAgo's higher moderate‐temperature cleavage activity.

### 2.6 Further Enhanced Catalytic Activity of mPfAgo and Application of This Engineering Strategy to TtdAgo

Given the enhanced high‐temperature activity of a previously described PfAgo mutant (I569Y/Y743F) through the alanine scanning strategy by Feng et al.,^[^ ^34^ ^]^ we further combined the identified mutation with the I569Y/Y743F mutation, generating a tetra‐mutant of PfAgo, called En‐mPfAgo (K617G/L618G/I569Y/Y743F). Functional analysis indicated higher cleavage activity of En‐mPfAgo toward both DNA and RNA targets than that of mPfAgo (Figure S8A, Supporting Information). Moreover, En‐mPfAgo also exhibited effective dsDNA cleavage activity in the presence of Mg^2+^ (Figure 4C). Meanwhile, a penta‐mutant (K617G/L618G/S578G/K612G/ D619G) was created as well, which showed DNA cleavage activity mediated by all four types of guides together with cleavage activity toward RNA target with three types of guides at 37 °C (Figure S8B, Supporting Information), indicating its higher substrate versatility than mPfAgo's. These results suggested that the K617G/L618G double mutation of PfAgo may play a pivotal role either in the promotion of enzymatic activity or in the magnification of functional diversity, which may facilitate generating more better‐than‐expected variants of PfAgo.

**Figure 6:**
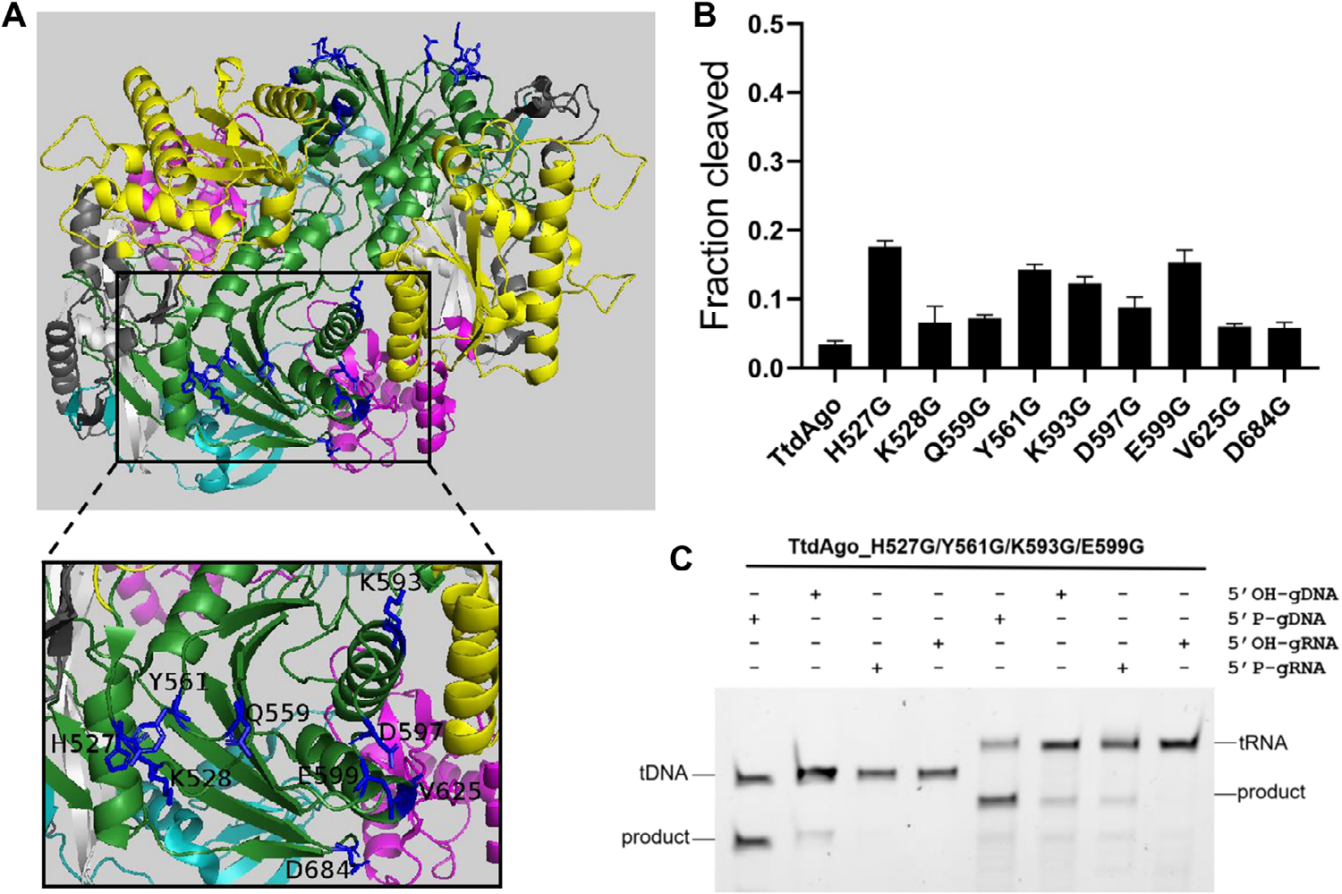
Rational engineering of TtdAgo generates mutants with increased activity at 37 °C and expanded substrate spectrum. A) Structure of TtdAgo ternary complex with the mutation sites marked. B) DNA cleavage activity assay of TtdAgo and its mutants at 37 °C (*n* = 3). Data are represented as the mean ± SD. C) Cleavage activity assay of the engineered TtdAgo_H527G/Y561G/K593G/E599G at 37 °C using 5′FAM‐labeled DNA and RNA as targets, respectively.

Additionally, this well‐established strategy for PfAgo's cold‐adaption engineering was successfully applied to another thermophilic archaea‐derived Ago protein, TtdAgo, yielding the engineered TtdAgo (H527G/Y561G/K593G/E599G) with improved activity and extended substrate spectrum at 37 °C (**Figure** **6**).

To explore the evolutionary conservation of these mutation sites, alignment of coding sequences between TtdAgo and PfAgo was conducted. The results showed that TtAgo shares 54.3% sequence identity with PfAgo. Besides, several residues of the mutation sites were found at homologous positions between these two Ago proteins, specifically the amino acids Y561, K593, D597, and E599 in TtdAgo correspond to the amino acids Y581, E613, K617, and D619 in PfAgo, respectively (Figure S9, Supporting Information). Given the enhanced moderate‐temperature cleavage activities of both TtdAgo and PfAgo resulting from the glycine substitution of each of these four homologous‐position residues, we speculated that these homologous sites may be of evolutionary importance in affecting the temperature‐dependent catalytic activities of either TtdAgo or PfAgo. Thus, these results would help our understanding of the evolution of Ago proteins’ temperature adaption, as well as guide the engineering of Ago proteins for variants with different temperature adaptions.

## 3 Conclusion

Here, for the first time, we successfully rationally designed a thermophilic Ago protein, PfAgo, for cold adaptation and obtained a better‐performing mutant, mPfAgo, which assumed a wider catalytic temperature range as well as an unexpectedly broader substrate spectrum with the gDNA‐ or gRNA‐mediated RNA cleavage activity acquired, relative to its wild type. Apart from that, the engineered mPfAgo boosted its catalytic activity at moderate and high temperatures, and maintained high thermostability as its wild type did, which would vastly expand its application scope.

Far‐UV CD spectra analysis and DSF measurement unraveled higher structural flexibility as well as more unfolded tertiary structure of mPfAgo at high temperatures whereas more stable configuration of mPfAgo binary complex at moderate temperatures, relative to wild‐type PfAgo. Further assessment of their applications in nucleic acid detection revealed significantly higher sensitivity of mPfAgo, as low as attomolar‐level detection limit in the presence of Mg^2+^. Moreover, this established engineering strategy was successfully applied to another thermophilic Ago protein (TtdAgo), yielding the engineered TtdAgo with improved activity and extended substrate spectrum at 37 °C and demonstrating the wide applicability of this rational design method.

The high thermostability and moderate‐temperature activity of mPfAgo imply its potential in the development of in vivo genome editing and RNA targeting tools. Agos with moderate‐temperature cleavage activities or desired substrate profiles are largely obtained through protein mining.^[^ ^35^, ^36^, ^37^, ^38^ ^]^ Hence, this study provides an alternate way to develop various Ago proteins with novel properties as well as a novel paradigm for other Ago's cold‐adaption engineering. Currently, residues proximity to the catalytic active center of Agos were paid concentrated attention for probing the catalytic mechanism of Agos, whereas less concern about the surface‐exposed residues.^[^ ^39^, ^40^ ^]^ Therefore, further research on the catalytic mechanism of mPfAgo is essential through diverse approaches, such as resolving the structures of mPfAgo at different temperatures, molecular dynamics simulation analysis about the differences in guide/target binding at different temperatures between PfAgo and mPfAgo, which would greatly advance our knowledge about Agos.

## 4 Experimental Section

### Structure‐Based Cold‐Adaptive Rational Design of PfAgo and TtdAgo

First, the ternary complex structure of PfAgo or TtdAgo was loaded into PyMOL software. Then, the six domains and bound DNA molecules of Ago proteins were marked with different colors, and amino acid residues around guide/target DNA and DEDH catalytic tetrad within 20Å were excluded. Next, the remaining surface‐exposed amino acid residues in the PIWI domain were chosen to be substituted with glycine, respectively. The PfAgo and TtdAgo genes were synthesized and codon‐optimized by Wuhan Genecreate, and inserted into the pET28a expression vector, respectively, in frame with the N‐terminal His tag. PfAgo and TtdAgo mutants were constructed based on T5 exonuclease I (NEB) and low‐temperature operation.

### Protein Expression and Purification

*Escherichia coli* strain BL21(DE3) was used as the host for protein expression. Single colonies were inoculated in LB liquid medium (5 mL) containing 50 mg mL^−1^ Kana (LK medium) for overnight growing at 37 °C, followed by transferring to LK medium (1 L) at a ratio of 1:5. When the OD_600_ value reached 0.6‐0.8, IPTG with the final concentration of 0.5 mm was added to induce protein expression at 18 °C for 16–20 h. The bacterial cells were collected by centrifuging at 6000 rpm for 10 min. The bacterial cells harvested were suspended in buffer A (20 mm Tris‐HCl pH 7.5, 400 mm NaCl, 5% (v/v) glycerol, 1 mm DTT, 1 mm PMSF) and lysed using a high‐pressure homogenizer, followed by centrifugation at 18 000 rpm for 50 min. The supernatant was filtered with a 0.45 µm filter membrane. The filtered supernatant was bound to Ni‐NTA agarose resin for 50 min at 4 °C. After washing with buffer A containing 20 and 50 mm imidazole, respectively, proteins were eluted using gradually increased concentration of imidazole in buffer A from 75 to 500 mm. The purified protein was analyzed using SDS‐PAGE and then concentrated by ultrafiltration using an Amicon 50K filter unit (Millipore). For TtdAgo and its mutants, the concentrated protein was applied to a 5‐mL Heparin HiTrap column (GE Life Sciences) for further purification. Protein concentration was determined using Bradford Protein Assay Kit (Beyotime), and the concentrated protein was flash‐frozen in liquid nitrogen and stored at −80 °C.

### Cleavage Assay of Single‐Stranded DNA and RNA

Guide and target nucleic acids, including 5′‐FAM‐labeled targets and 5′‐P/COOH‐labeled guides, were synthesized by Sangon and Genscript (Tables S1, Supporting Information). Unless otherwise indicated, purified proteins, guides, and targets were generally mixed with a 4:4:1 ratio (800 nm protein: 800 nm guide: 200 nm target) in reaction buffer (10 mm HEPES‐NaOH pH 8.0, 100 mm NaCl, 5 mm MnCl_2_, 5% glycerol). Protein was first premixed with gDNA or gRNA for 10 min at 37 °C for guide loading. Then target DNA or RNA was added and incubated for 30 min at 37 °C for cleavage. To investigate the effects of different divalent metal ions on the cleavage activity, MnCl_2_ in the reaction buffer was replaced by FeCl_2_, CoCl_2_, NiCl_2_, CuCl_2_, ZnCl_2_, CaCl_2_, MgCl_2,_ or EDTA, respectively. For effects of pH or NaCl concentration on the cleavage activity, reaction buffer with different pH (6.5–9) or NaCl concentration (0–200 mm) was applied to cleavage reaction. The cleavage kinetics were determined through time‐course cleavage of target (0–60 min) at 37 °C. The heat resistance of mPfAgo was examined by premixing with 5′P‐gDNA for 30 min at 37–90 °C, respectively, and the following addition of target DNA for cleavage. The temperature dependence of target cleavage was analyzed by incubating the samples at indicated temperatures using a PCR thermocycler (Bio‐Rad). The substrate turnover kinetics of mPfAgo or PfAgo were determined by premixing different concentrations (50–800 nm) of protein with 5′P‐gDNA respectively for guide loading, and the following addition of target DNA for cleavage. All reactions were terminated by adding an equal volume of RNA loading dye (95% formamide, 18 mm EDTA, 0.025% SDS, and 0.025% bromophenol blue) and heating for 5 min at 95 °C. Cleavage products were resolved on 20% denaturing polyacrylamide gel and visualized using Gel Doc XR+ (Bio‐Rad). The parameters *k*_cat_ and *K*_M_ of PfAgo or mPfAgo were determined by fitting the Michaelis–Menten equation to the velocity of each reaction as a function of the concentration of target DNA. Here, 8 µm 5′P‐gDNA, 2 µM protein, and eight different concentrations of target DNA ranging from 0.5 to 8 µm were used. The cleavage rate of PfAgo or mPfAgo was determined according to the maximum slope of the fluorescence intensity‐time curve. For the cleavage activity assays of mPfAgo in bacterial or mammalian cell lysates, *Escherichia coli* cells were lysed using lysozyme (Solarbio) in a water bath at 37 °C for 30 min, and HEK293T cells were lysed using RIPA lysis buffer (Beyotime) at room temperature for 15 min. Subsequently, 2 µL of the respective lysates were added to the mPfAgo reaction system and reacted at 37 °C for 45 min.

### Cleavage Assay of Double‐Stranded DNA

The 1 kb‐long dsDNA was amplified from pUC19 with forward primer (5′‐TTTCCCCCTGGAAGCTCCCTCGTGCG‐3′) and reverse primer (5′‐CCTGTAGCAATGGCAACAACGTTGCGC‐3′). The gDNA sequences used in dsDNA cleavage were shown in Table S2 (Supporting Information). 0.5 µM mPfAgo was premixed with 1 µM forward or reverse guides respectively in reaction buffer containing 10 mm HEPES‐NaOH (pH 8.0), 100 mm NaCl, 0.5 mm MnCl_2_/2.5 mm MgCl_2_ and 5% glycerol for 10 min at 70 °C for guide loading. Then the two separate reaction mixtures were mixed followed by addition of dsDNA (200 ng) and incubation for 5 min at 95 °C. The cleavage products were mixed with DNA loading dye (NEB) followed by 1% agarose gel electrophoresis, EB staining, and visualization using Gel Doc XR+ (Bio‐Rad).

### PfAgo/mPfAgo‐Mediated Nucleic Acid Detection

The DNA sequences used in nucleic acid detection were shown in Table S3 (Supporting Information). The E6 gene from HPV16 and the *swp* gene from *Enterocytozoon hepatopenaei* were inserted into the pUC19 vector, respectively. PCR was carried out for 30 thermal cycles (95 °C for 10 s, 55 °C for 10 s, and 72 °C for 5 s) in a volume of 10 µL, which contained 1×Taq DNA polymerase Mix (Tsingke), 0.5 µm F/R‐primers, and different amounts of target pUC19‐E6. 5 µL of PCR products were transferred to the cleavage reaction mixture with the volume of 10 µL, containing 1× PfAgo reaction buffer (10 mM HEPES pH 7.5, 50 mm NaCl, and 10 mm MgCl_2_), 2 µg PfAgo or mPfAgo, 0.5 µm gDNA and 1 µm molecular beacon. RPA was performed using RAA Nucleic Acids Amplification Kit (ZC Bioscience) according to the manufacturer's instructions. Similarly, 2 µL of RPA products were transferred to the cleavage reaction mixture with the volume of 10 µL, containing 1×PfAgo reaction buffer, 1 µg PfAgo or mPfAgo, 0.5 µm gDNA and 1 µm molecular beacon. After incubation at 90 °C for 30 or 20 min, the fluorescence intensity was detected using Real‐time fluorescence quantitative PCR instrument (Bio‐Rad).

### CD Spectroscopy

The CD measurement on the evolution of the secondary structure of PfAgo or mPfAgo was performed in a CD spectrometer (Applied Photophysics) with a 10 mm pathlength cell. The concentration of PfAgo or mPfAgo was 0.2 mg mL^−1^ in PBS buffer (pH 8.0). The wavelength range of detection was 190–260 nm. PfAgo or mPfAgo was heated in the range of 25 to 100 °C with a 1 °C min^−1^ heating rate and equilibrated for 1 min at each temperature, and the CD data were collected at 1 °C intervals. The results at 37, 45, 55, and 95 °C were analyzed using CDNN software. The signals in the far‐UV CD region (222 nm) were monitored as a function of temperature to determine the thermal unfolding of PfAgo or mPfAgo.

### DSF

2 µm of PfAgo or mPfAgo in a buffer containing 15 mm Tris‐HCl (pH 8.0) and 250 mm NaCl was prepared in triplicate and added to PCR tubes. SYPRO Orange dye available as 5000× stock (Sigma‐Aldrich) was added just before the measurement in an appropriate amount to achieve a final concentration of the dye of 5×. The thermal denaturation of PfAgo or mPfAgo was monitored by exciting the SYPRO Orange dye at 470 nm and monitoring its fluorescence emission at 570 nm using Real‐time fluorescence quantitative PCR instrument (Bio‐Rad). The DSF experiments for PfAgo/mPfAgo binary and ternary complexes were performed in the presence of 5 mm Mg^2+^.

### Fluorescence Polarization Assay

Fluorescence polarization assays to measure the interactions of proteins with nucleic acids were performed using a multifunctional microplate reader (TECAN). To analyze the affinity of PfAgo and mPfAgo for 5′P‐gDNA, PfAgo and mPfAgo were incubated with 5′P‐gDNA, respectively, in 100 µL of binding buffer containing 10 mm HEPES‐NaOH (pH 7.5), 100 mm NaCl, 5 mm MnCl_2_, and 5% glycerol for 1 h at 37 °C. The guides used in the experiments were 3′‐FAM‐labeled. The concentration of guides was fixed at 2.5 nm, whereas the concentration of PfAgo and mPfAgo varied. After incubation, samples were applied to fluorescence polarization assay. To analyze the affinity of PfAgo and mPfAgo for the 5′P‐gDNA:target DNA duplexes, guides, and targets were first annealed to form duplexes by heating at 95 °C for 2 min, then decreasing to 25 °C at a rate of 0.1 °C s^−1^ and finally maintaining for 2 min. Then PfAgo_DM and mPfAgo_DM were incubated with the 5′P‐gDNA: target DNA duplex, respectively, in 100 µL of binding buffer containing 10 mm HEPES‐NaOH (pH 7.5), 100 mm NaCl, 5 mm MnCl_2_, and 5% glycerol for 1 h at 37 °C. The targets used in the experiments were 5′‐FAM‐labeled. The concentration of nucleic acid duplexes was fixed at 2.5 nm, whereas the protein concentration varied. After incubation, the samples were applied to fluorescence polarization assay.

### Statistical Analysis

ImageJ, Excel (Office), and Prism 8 (GraphPad) software were used for statistical analyses and graph generation. All experiments were repeated at least three times. Data were expressed as the mean ± standard deviation (SD). For comparisons between two groups, the *t*‐test assuming a two‐tailed distribution was employed. Significance was defined as #*p* > 0.05, *p < 0.05, **p < 0.01, ***p < 0.001, and ****p < 0.0001.

## Conflict of Interest

L.M., L.W., F.W., X.X., W.C., and F.H. are listed as inventors on an awarded patent (US012054757B1) concerning the design and application of PfAgo mutants. All other authors declared no conflict of interest.

## Supporting information



Supporting Information

## Data Availability

The data that support the findings of this study are available from the corresponding author upon reasonable request.
