# Supplementary material for: An Engineered PfAgo with Wide Catalytic Temperature Range and Substrate Spectrum
Source: Adv Sci (Weinh). 2025 May 14;12(29):2416631. doi: 10.1002/advs.202416631 (PMC12362761; doi:10.1002/advs.202416631)
Supplement: Supplementary file 1 — Supporting Information [file ADVS-12-2416631-s001.pdf]

ADVANCED SCIENCE
Open Access

Supporting Information

for *Adv. Sci.*, DOI 10.1002/advs.202416631

An Engineered PfAgo with Wide Catalytic Temperature Range and Substrate Spectrum

Longyu Wang, Xiaochen Xie, Fuyong Huang, Qiang Wei, Tianxin Cai, Na Yu, Shi Chen, Fei Wang, Wanping Chen, Chin-yu Chen, Chunhua Li and Lixin Ma**

Supporting Information

An Engineered PfAgo with Wide Catalytic Temperature Range and Substrate Spectrum

Longyu Wang, Xiaochen Xie, Fuyong Huang, Qiang Wei, Tianxin Cai, Na Yu, Shi Chen, Fei Wang, Wanping Chen, Chin-yu Chen, Chunhua Li, and Lixin Ma**

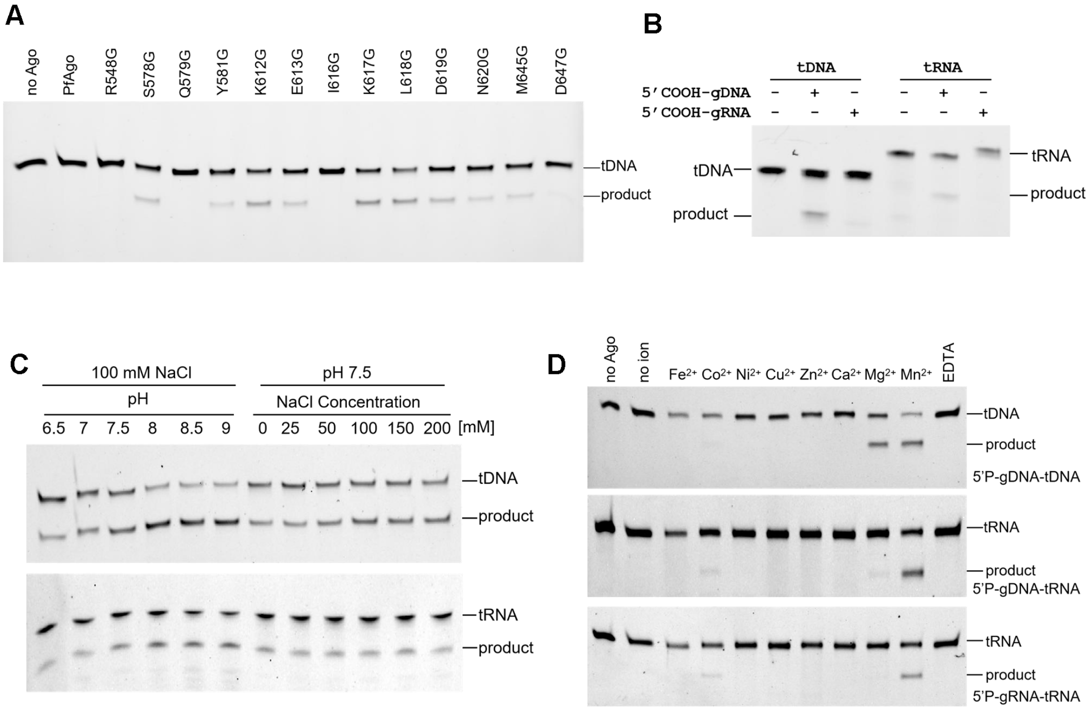

Figure S1. Cleavage activity assay of PfAgo and its mutants at 37 °C.

(A) The gel diagram showing 5’P-gDNA-mediated DNA cleavage by PfAgo and its single mutants at 37 °C, respectively.

(B) The gel diagram showing target DNA and RNA cleavage activity of mPfAgo using 5’COOH-gDNA or 5’COOH-gRNA at 37 °C.

(C) The gel diagram showing the activity of mPfAgo at 37 °C under different pH or NaCl concentrations.

(D) The gel diagram showing the cleavage activity of mPfAgo at 37 °C in the presence of different divalent metal ions.

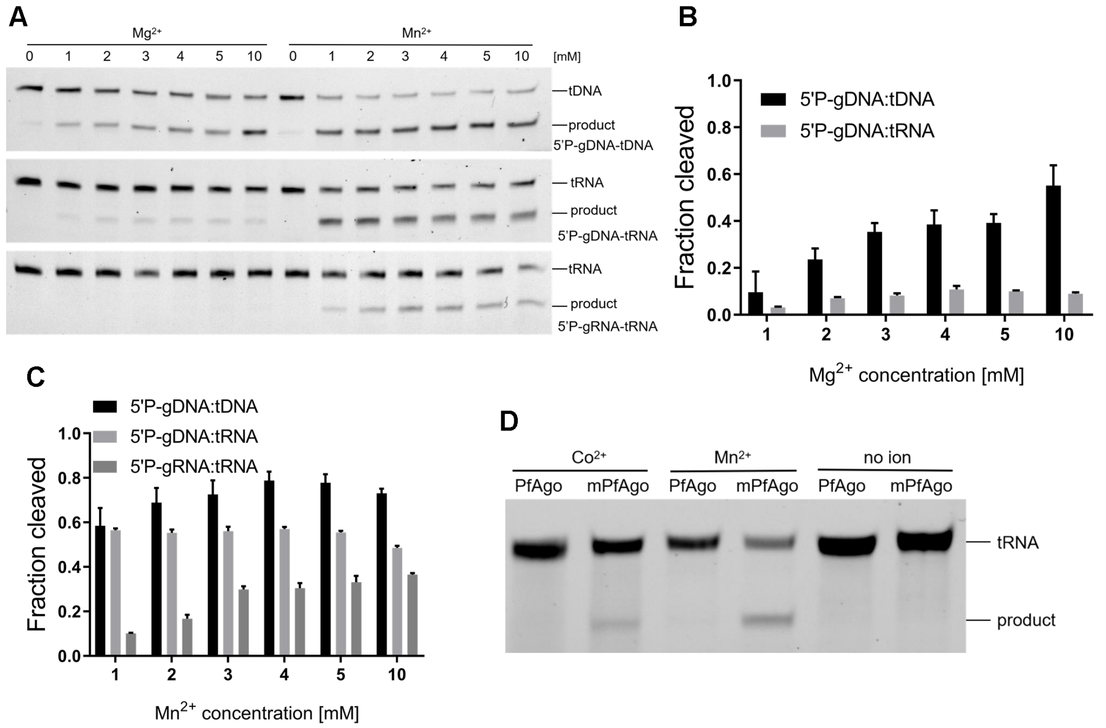

Figure S2. Effects of divalent metal ions on the cleavage activity.

(A) The gel diagram showing mPfAgo activity at 37 °C at different Mg^{2+} or Mn^{2+} concentrations.

(B) Effects of Mg^{2+} concentration on the mPfAgo cleavage activity were inspected, respectively, at 37 °C (*n*=3). Data are represented as the mean ± SD.

(C) Effects of Mn^{2+} concentration on the mPfAgo cleavage activity were inspected, respectively, at 37 °C (*n*=3). Data are represented as the mean ± SD.

(D) The gel diagram showing 5'P-gRNA-mediated RNA cleavage at 80 °C by PfAgo and mPfAgo in the presence of Mg^{2+} or Mn^{2+}.

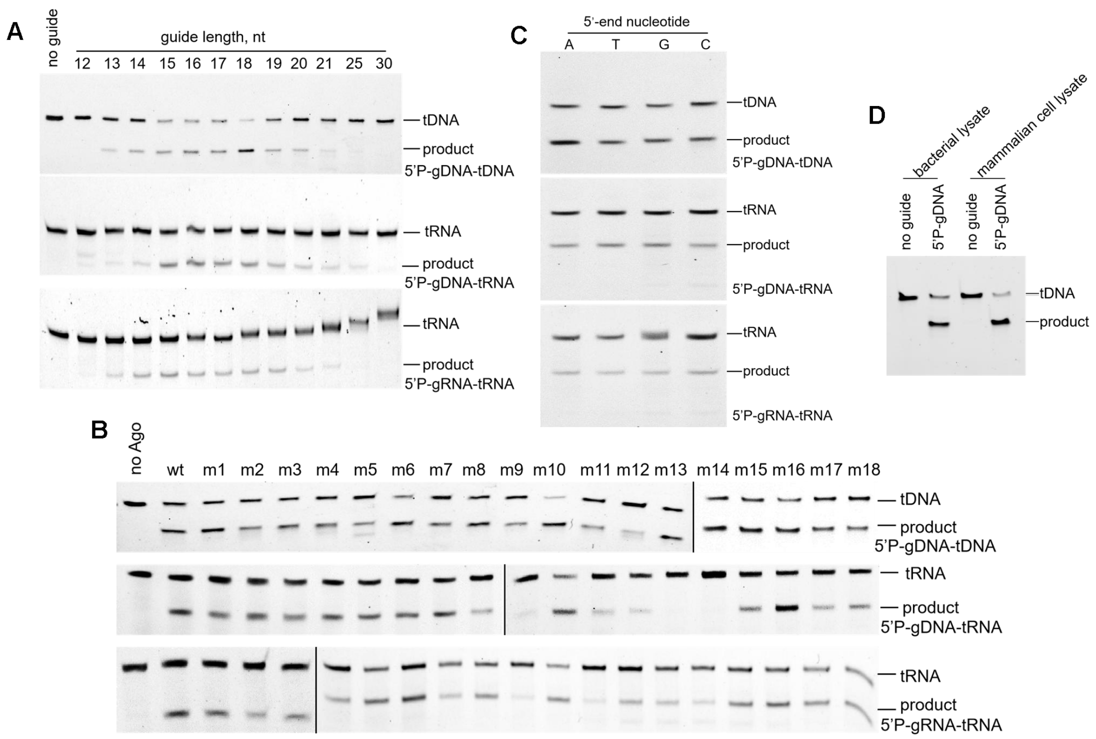

Figure S3. Cleavage activity assay of mPfAgo with varied guide lengths and sequences.

(A) The gel diagram showing mPfAgo activity at 37 ℃ using guides of varied lengths.

(B) The gel diagram showing mPfAgo activity at 37 ℃ with different guide-target mismatch sites.

(C) The gel diagram showing mPfAgo activity at 37 ℃ using guides with different 5'-end nucleotides.

(D) The gel diagram showing 5'P-gDNA-mediated DNA cleavage at 37 ℃ by mPfAgo in bacterial and mammalian cell lysates.

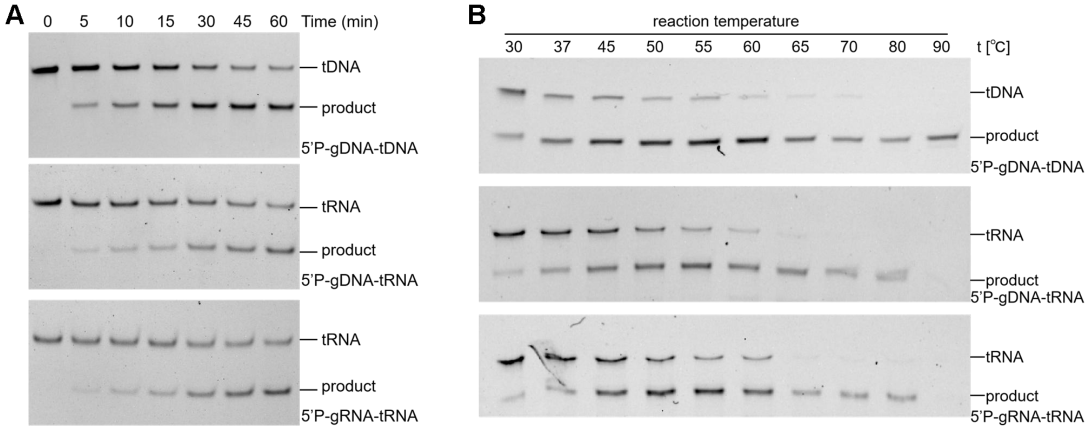

Figure S4. Assay of cleavage activity of mPfAgo at different times and temperatures.

(A) The gel diagram showing mPfAgo cleavage activity at different temperatures.

(B) The gel diagram showing the time-course of target cleavage at 37 ℃ by mPfAgo.

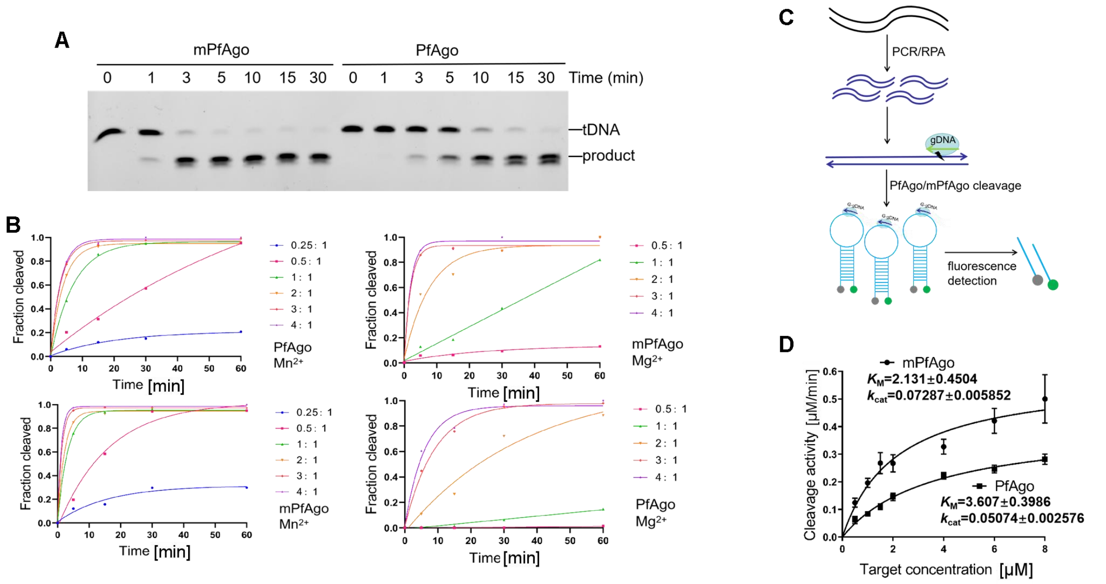

Figure S5. Assay of cleavage activity of PfAgo and mPfAgo at high temperatures.

(A) The gel diagram showing the time-course of target DNA cleavage at 95 °C by mPfAgo or PfAgo in the presence of Mg^{2+}.

(B) Turnover rate analysis of PfAgo and mPfAgo through 5'P-DNA-guided DNA cleavage at 95 °C with varied ratios of Ago/gDNA to tDNA in the presence of Mn^{2+} or Mg^{2+}.

(C) Schematic diagram of PfAgo/mPfAgo-based nucleic acid detection.

(D) Michaelis-Menten kinetics analysis of PfAgo and mPfAgo at 95 °C (*n*=3). Data are represented as the mean ± SD.

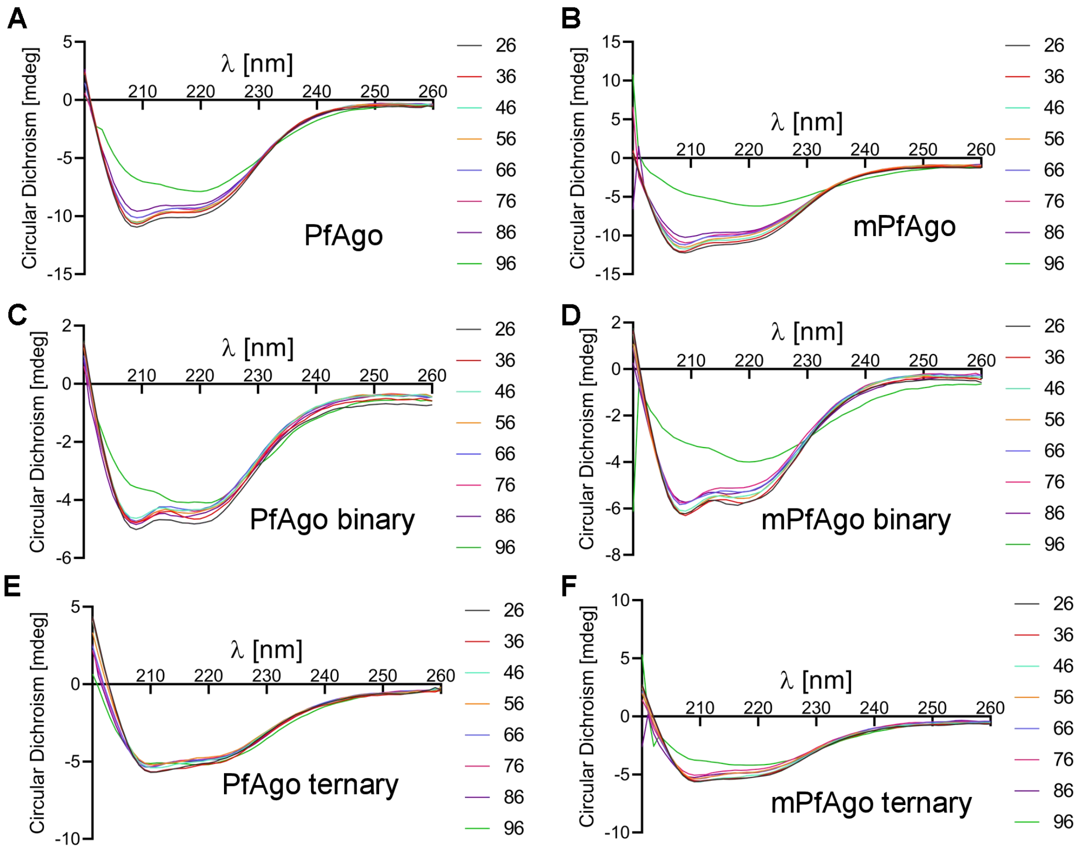

Figure S6. Analysis of secondary structures of PfAgo and mPfAgo at distinct temperatures using far-UV CD spectroscopy.

Far-UV CD spectra of PfAgo (A), mPfAgo (B), PfAgo binary complex (C), mPfAgo binary complex (D), PfAgo ternary complex (E), and mPfAgo ternary complex (F) were measured at different temperatures.

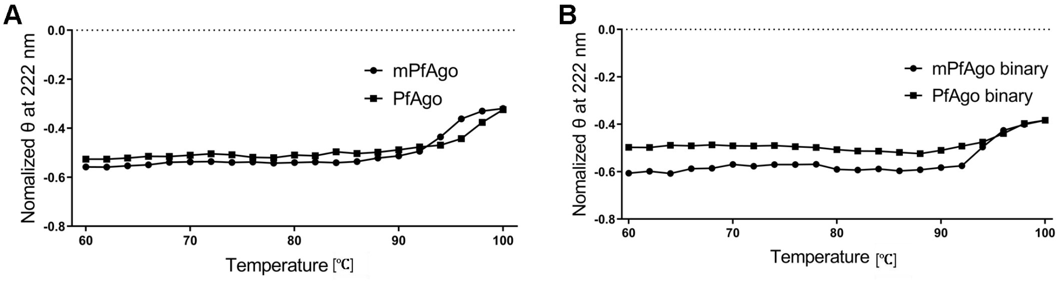

C

	PfAgo	mPfAgo	PfAgo Binary	mPfAgo Binary	PfAgo Ternary	mPfAgo Ternary
T_m (℃)	90.77-91.56	87.73-88.38	91.05-92.23	88.90-89.69	92.24-93.25	90.54-91.47

Figure S7. Thermal unfolding curves and T_m values of PfAgo and mPfAgo. Thermal unfolding curves of PfAgo/mPfAgo (A) and their binary complexes (B) were measured using far-UV CD spectroscopy. (C) The respective T_m values of PfAgo and mPfAgo were calculated via DSF measurement.

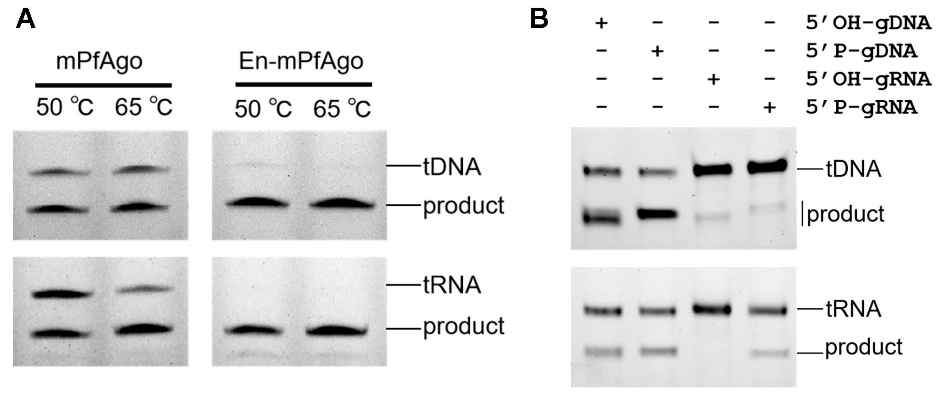

Figure S8. Cleavage activity assay of PfAgo mutants.

(A) Cleavage activity assay of mPfAgo and En-mPfAgo (K617G/L618G/I569Y/Y743F). The cleavage time was 20 minutes.

(B) Cleavage activity assay of the PfAgo penta-mutant (K617G/L618G/S578G/K612G/D619G) at 37 ℃.

A

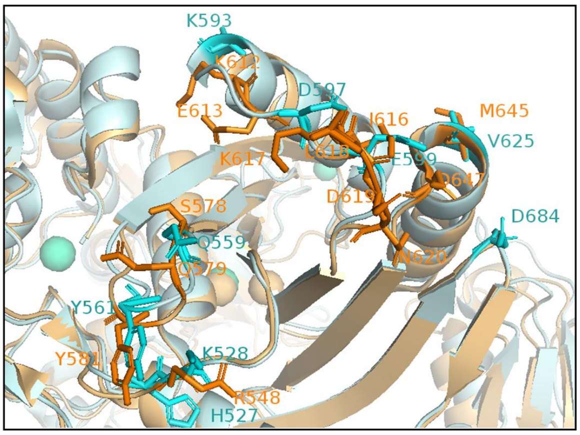

B

```
PfAgo  VLSKLGVKYYVLDYRFNYDYIIGIDVAPMKRSEGYIGGSAV
TtdAgo ILSKLGIKYYVLRHKFSYDYIVGIDVTPMKLSHGYIGGSAV

PfAgo  MFDSQ-GYIRKIVPIKIGEQRGESVDMNEFF---KEMVDKF
TtdAgo MFDSQ-GYIRKIIPVEIGEQMGESIDMKEFF---KDMVVQF

PfAgo  KEFNIKLDNKKILLLRDGRITNNEEEGLKYISEMFDIE 649
TtdAgo GKFGIDLEGKSILILRDGKITKDEEEGLAYISKVFGIK 629
```

Figure S9. Sequence comparison of PfAgo and TtdAgo.

(A) PfAgo (8jpx, orange) and TtdAgo (8wd8, cyan) were aligned using their PIWI domains. The mutation sites of PfAgo and TtdAgo are marked with orange and cyan, respectively.

(B) Structure-based sequence alignment of PIWI-domains between PfAgo and TtdAgo. The mutated amino acids are highlighted in yellow.

Table S1 The nucleic sequences used in ssDNA and RNA cleavage.

Oligonucleotide name	Sequence (5'-3')	Description
FAM-A-tDNA	FAM-AAACGACGGCCAGTGCCAAG CTTACTATACAACCTACTACCTCTT	5’FAM labeled A-tDNA
FAM-T-tDNA	FAM-AAACGACGGCCAGTGCCAAG CTTACTATACAACCTACTACCTCAT	5’FAM labeled T-tDNA
FAM-G-tDNA	FAM-AAACGACGGCCAGTGCCAAG CTTACTATACAACCTACTACCTCCT	5’FAM labeled G-tDNA
FAM-C-tDNA	FAM-AAACGACGGCCAGTGCCAAG CTTACTATACAACCTACTACCTCGT	5’FAM labeled C-tDNA
FAM-A-tRNA	FAM-AAACGACGGCCAGUGCCAAG CUUACUAUACAACCUACUACCUCUU	5’FAM labeled A-tRNA
FAM-U-tRNA	FAM-AAACGACGGCCAGUGCCAAG CUUACUAUACAACCUACUACCUCAU	5’FAM labeled U-tRNA
FAM-G-tRNA	FAM-AAACGACGGCCAGUGCCAAG CUUACUAUACAACCUACUACCUCCU	5’FAM labeled G-tRNA
FAM-C-tRNA	FAM- AAACGACGGCCAGUGCCAAG CUUACUAUACAACCUACUACCUCGU	5’FAM labeled C-tRNA
A-gDNA	AGAGGTAGTAGGTTGTAT	DNA guide forms 5'-A pair with A-tDNA/A-tRNA
T-gDNA	TGAGGTAGTAGGTTGTAT	DNA guide forms 5'-T pair with T-tDNA/T-tRNA
G-gDNA	GGAGGTAGTAGGTTGTAT	DNA guide forms 5'-G pair with G-tDNA/G-tRNA
C-gDNA	CGAGGTAGTAGGTTGTAT	DNA guide forms 5'-C pair with C-tDNA/C-tRNA
A-gRNA	AGAGGUAGUAGGUUGUAU	RNA guide forms 5'-A pair with A-tDNA/A-tRNA
U-gRNA	UGAGGUAGUAGGUUGUAU	RNA guide forms 5'-U pair with T-tDNA/U-tRNA
G-gRNA	GGAGGUAGUAGGUUGUAU	RNA guide forms 5'-G pair with G-tDNA/G-tRNA
C-gRNA	CGAGGUAGUAGGUUGUAU	RNA guide forms 5'-C pair with C-tDNA/C-tRNA
M1	FAM-AAACGACGGCCAGTG CCAAGCTTACTATACAACC	5’ FAM labeled 34 nt DNA
M2	FAM-AAACGACGGCCAGUG CCAAGCUUACUAUACAACC	5’ FAM labeled 34 nt RNA
gDNA_mm1	AGAGGTAGTAGGTTGTAT	DNA guide forms mismatched pair in position 1 with tDNA/tRNA

gDNA_mm2	TCAGGTAGTAGGTTGTAT	DNA guide forms mismatched pair in position 2 with tDNA/tRNA
gDNA_mm3	TGTGGTAGTAGGTTGTAT	DNA guide forms mismatched pair in position 3 with tDNA/tRNA
gDNA_mm4	TGACGTAGTAGGTTGTAT	DNA guide forms mismatched pair in position 4 with tDNA/tRNA
gDNA_mm5	TGAGCTAGTAGGTTGTAT	DNA guide forms mismatched pair in position 5 with tDNA/tRNA
gDNA_mm6	TGAGGAAGTAGGTTGTAT	DNA guide forms mismatched pair in position 6 with tDNA/tRNA
gDNA_mm7	TGAGGTTGTAGGTTGTAT	DNA guide forms mismatched pair in position 7 with tDNA/tRNA
gDNA_mm8	TGAGGTACTAGGTTGTAT	DNA guide forms mismatched pair in position 8 with tDNA/tRNA
gDNA_mm9	TGAGGTAGAAGGTTGTAT	DNA guide forms mismatched pair in position 9 with tDNA/tRNA
gDNA_mm10	TGAGGTAGTTGGTTGTAT	DNA guide forms mismatched pair in position 10 with tDNA/tRNA
gDNA_mm11	TGAGGTAGTACGTTGTAT	DNA guide forms mismatched pair in position 11 with tDNA/tRNA
gDNA_mm12	TGAGGTAGTAGCTTGTAT	DNA guide forms mismatched pair in position 12 with tDNA/tRNA
gDNA_mm13	TGAGGTAGTAGGATGTAT	DNA guide forms mismatched pair in position 13 with tDNA/tRNA
gDNA_mm14	TGAGGTAGTAGGTAGTAT	DNA guide forms mismatched pair in position 14 with tDNA/tRNA
gDNA_mm15	TGAGGTAGTAGGTTCTAT	DNA guide forms mismatched pair in position 15 with tDNA/tRNA
gDNA_mm16	TGAGGTAGTAGGTTGAAT	DNA guide forms mismatched

		pair in position 16 with tDNA/tRNA
gDNA_mm17	TGAGGTAGTAGGTTGTTT	DNA guide forms mismatched pair in position 17 with tDNA/tRNA
gDNA_mm18	TGAGGTAGTAGGTTGTAA	DNA guide forms mismatched pair in position 18 with tDNA/tRNA
gRNA_mm1	AGAGGUAGUAGGUUGUAU	RNA guide forms mismatched pair in position 1 with tDNA/tRNA
gRNA_mm2	UCAGGUAGUAGGUUGUAU	RNA guide forms mismatched pair in position 2 with tDNA/tRNA
gRNA_mm3	UGUGGUAGUAGGUUGUAU	RNA guide forms mismatched pair in position 3 with tDNA/tRNA
gRNA_mm4	UGACGUAGUAGGUUGUAU	RNA guide forms mismatched pair in position 4 with tDNA/tRNA
gRNA_mm5	UGAGCUAGUAGGUUGUAU	RNA guide forms mismatched pair in position 5 with tDNA/tRNA
gRNA_mm6	UGAGGAAGUAGGUUGUAU	RNA guide forms mismatched pair in position 6 with tDNA/tRNA
gRNA_mm7	UGAGGUUGUAGGUUGUAU	RNA guide forms mismatched pair in position 7 with tDNA/tRNA
gRNA_mm8	UGAGGUACUAGGUUGUAU	RNA guide forms mismatched pair in position 8 with tDNA/tRNA
gRNA_mm9	UGAGGUAGAAGGUUGUAU	RNA guide forms mismatched pair in position 9 with tDNA/tRNA
gRNA_mm10	UGAGGUAGUUGGUUGUAU	RNA guide forms mismatched pair in position 10 with tDNA/tRNA
gRNA_mm11	UGAGGUAGUACGUUGUAU	RNA guide forms mismatched pair in position 11 with tDNA/tRNA
gRNA_mm12	UGAGGUAGUAGCUUGUAU	RNA guide forms mismatched pair in position 12 with

		tDNA/tRNA
gRNA_mm13	UGAGGUAGUAGGAUGUAU	RNA guide forms mismatched pair in position 13 with tDNA/tRNA
gRNA_mm14	UGAGGUAGUAGGUAGUAU	RNA guide forms mismatched pair in position 14 with tDNA/tRNA
gRNA_mm15	UGAGGUAGUAGGUUCUAU	RNA guide forms mismatched pair in position 15 with tDNA/tRNA
gRNA_mm16	UGAGGUAGUAGGUUGAAU	RNA guide forms mismatched pair in position 16 with tDNA/tRNA
gRNA_mm17	UGAGGUAGUAGGUUGUUU	RNA guide forms mismatched pair in position 17 with tDNA/tRNA
gRNA_mm18	UGAGGUAGUAGGUUGUAA	RNA guide forms mismatched pair in position 18 with tDNA/tRNA
12nt T-gDNA	TGAGGTAGTAGG	12 nt DNA guide pair with T-tDNA/U-tRNA
13nt T-gDNA	TGAGGTAGTAGGT	13 nt DNA guide pair with T-tDNA/U-tRNA
14nt T-gDNA	TGAGGTAGTAGGTT	14 nt DNA guide pair with T-tDNA/U-tRNA
15nt T-gDNA	TGAGGTAGTAGGTTG	15 nt DNA guide pair with T-tDNA/U-tRNA
16nt T-gDNA	TGAGGTAGTAGGTTGT	16 nt DNA guide pair with T-tDNA/U-tRNA
17nt T-gDNA	TGAGGTAGTAGGTTGTA	17 nt DNA guide pair with T-tDNA/U-tRNA
19nt T-gDNA	TGAGGTAGTAGGTTGTATA	19 nt DNA guide pair with T-tDNA/U-tRNA
20nt T-gDNA	TGAGGTAGTAGGTTGTATAG	20 nt DNA guide pair with T-tDNA/U-tRNA
21nt T-gDNA	TGAGGTAGTAGGTTGTATAGT	21 nt DNA guide pair with T-tDNA/U-tRNA
25nt T-gDNA	TGAGGTAGTAGGTTGTAT AGTAAGC	25 nt DNA guide pair with T-tDNA/U-tRNA
30nt T-gDNA	TGAGGTAGTAGGTTGTAT AGTAAGCTTGGC	30 nt DNA guide pair with T-tDNA/U-tRNA
12nt U-gRNA	UGAGGUAGUAGG	12 nt RNA guide pair with T-tDNA/U-tRNA

13nt U-gRNA	UGAGGUAGUAGGU	13 nt RNA guide pair with T-tDNA/U-tRNA
14nt U-gRNA	UGAGGUAGUAGGUU	14 nt RNA guide pair with T-tDNA/U-tRNA
15nt U-gRNA	UGAGGUAGUAGGUUG	15 nt RNA guide pair with T-tDNA/U-tRNA
16nt U-gRNA	UGAGGUAGUAGGUUGU	16 nt RNA guide pair with T-tDNA/U-tRNA
17nt U-gRNA	UGAGGUAGUAGGUGUA	17 nt RNA guide pair with T-tDNA/U-tRNA
19nt U-gRNA	UGAGGUAGUAGGUUGUAUA	19 nt RNA guide pair with T-tDNA/U-tRNA
20nt U-gRNA	UGAGGUAGUAGGUUGUAUAG	20 nt RNA guide pair with T-tDNA/U-tRNA
21nt U-gRNA	UGAGGUAGUAGGUUGUAUAGU	21 nt RNA guide pair with T-tDNA/U-tRNA
25nt U-gRNA	UGAGGUAGUAGGUUGUAU AGUAAGC	25 nt RNA guide pair with T-tDNA/U-tRNA
30nt U-gRNA	UGAGGUAGUAGGUUGUAU AGUAAGCUUGGC	30 nt RNA guide pair with T-tDNA/U-tRNA

Table S2 The gDNA sequences used in dsDNA cleavage.

gDNA	Sequence (5'-3')	Description
gDNA-F	ATCTTCACCTAGATCC	DNA guide complementary with top strand of dsDNA
gDNA-R	AAAAGGATCTAGGTGA	DNA guide complementary with bottom strand of dsDNA

Table S3 The DNA sequences used in nucleic acid detection.

Oligonucleotide name	Sequence (5'-3')	Description
HPV16E6	ACGTCTAAGAAACCATTATTATCAT GACATACTGCAATGTTTCAGGACC CACAGGAGCGACCCAGAAAGTTA CCACAGTTATGCACAGAGCTGCA AACAACTATACATGATATAATATTAG AATGTGTGTACTGCAAGCAACAGT TACTGCGACGTGAGGTATATGACT TTGCTTTTCGGAGCTGCATTAATG AATCGGCCAACGCGCGG	E6 gene of HPV16
F-primer	ACGTCTAAGAAACCATTATTATCAT GACATACT	Forward primer of HPV16E6

R-primer	CCGCGCGTTGGCCGATTC	Reverse primer of HPV16E6
16-gDNA	GCCGATTCATTAATGCAG	Input guide DNA complementary with HPV16E6
PCR-MB	5'FAM-cgcaccCCGCGCGTTGGCCGATTCATggtgcg-3'BHQ	Molecular beacon complementary with newly generated gDNA from HPV16E6
swp	CAGCTTAAAGAAGTTTGCAATGATTTTTCTAAAGCATAGCATATGAATGCATATCAGAAGATAAAAGGAAAATG	*swp* gene of *Enterocytozoon hepatopenaei*
RPA-F	CAGCTTAAAGAAGTTTGCAATG	Forward primer of *swp*
RPA-R	CATTTTCCTTTTATCTTCTGATATGC	Reverse primer of *swp*
RPA-gDNA	CTTCTGATATGCATTC	Input guide DNA complementary with *swp* gene
RPA-MB	5'FAM-CGCACCCATTTTCCTTTTATCTTCTGATATGGTGCG-3'BHQ	Molecular beacon complementary with newly generated gDNA from *swp* gene
